# *N*-{*N*-[*N*-(1,1-Dimethyl­ethoxy­carbon­yl)-l-leuc­yl]-*N*-methyl-l-leuc­yl}-*N*-methyl-l-leucine benzyl ester

**DOI:** 10.1107/S1600536808034247

**Published:** 2008-10-25

**Authors:** Wen Jie Xu, Xiao Jian Liao, Jian Zhong Diao, Lei Zhou, Shi Hai Xu

**Affiliations:** a Institute of Hydrobiology, Jinan University, Guangzhou, Guangdong 510632, People’s Republic of China; b Department of Chemistry, Jinan University, Guangzhou, Guangdong 510632, People’s Republic of China

## Abstract

The tripeptide title compound, C_32_H_53_N_3_O_6_, synthesized in 80% yield by coupling of *N*-methyl-l-leucine benzyl ester with *tert*-butoxy­carbonyl-l-leucyl-*N*-methyl-l-leucine at 273 K, conjugates through two amide linkages and includes two protecting groups: a *tert*-butyl­oxycarbonyl group at the C-tip and a benzyl group at the N-tip. A classical inter­molecular N—H⋯O hydrogen bond and a weak non-conventional inter­molecular C—H⋯O contact connect the mol­ecules, forming layers parallel to (001).

## Related literature

For the structure of a related dipeptide, see: Liao *et al.* (2007 ▶). For the synthesis of linear peptide fragments of cyclic penta­peptide, see: Xu *et al.* (2008 ▶).
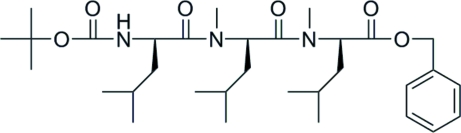


## Experimental

### 

#### Crystal data

C_32_H_53_N_3_O_6_
*M* *_r_* = 575.77
Trigonal, 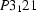

*a* = 13.9784 (3) Å
*c* = 30.4763 (15) Å
*V* = 5157.1 (3) Å^3^
*Z* = 6
Mo *K*α radiation
μ = 0.08 mm^−1^
*T* = 173 (2) K
0.47 × 0.42 × 0.26 mm

#### Data collection

Bruker SMART 1000 CCD diffractometer
Absorption correction: multi-scan (*SADABS*; Sheldrick, 1996 ▶) *T*_min_ = 0.965, *T*_max_ = 0.981
27507 measured reflections
4232 independent reflections
3541 reflections with *I* > 2σ(*I*)
*R*_int_ = 0.032

#### Refinement

*R*[*F* ^2^ > 2σ(*F* ^2^)] = 0.048
*wR*(*F* ^2^) = 0.138
*S* = 1.05
4232 reflections
381 parameters
18 restraints
H-atom parameters constrained
Δρ_max_ = 0.43 e Å^−3^
Δρ_min_ = −0.23 e Å^−3^

### 

Data collection: *SMART* (Bruker, 1997 ▶); cell refinement: *SAINT* (Bruker, 1997 ▶); data reduction: *SAINT*; program(s) used to solve structure: *SHELXS97* (Sheldrick, 2008 ▶); program(s) used to refine structure: *SHELXL97* (Sheldrick, 2008 ▶); molecular graphics: *SHELXTL* (Sheldrick, 2008 ▶); software used to prepare material for publication: *SHELXTL* and *PLATON* (Spek, 2003 ▶).

## Supplementary Material

Crystal structure: contains datablocks I, global. DOI: 10.1107/S1600536808034247/si2119sup1.cif

Structure factors: contains datablocks I. DOI: 10.1107/S1600536808034247/si2119Isup2.hkl

Additional supplementary materials:  crystallographic information; 3D view; checkCIF report

## Figures and Tables

**Table 1:** Hydrogen-bond geometry (Å, °)

*D*—H⋯*A*	*D*—H	H⋯*A*	*D*⋯*A*	*D*—H⋯*A*
N3—H3*A*⋯O3^i^	0.88	2.09	2.923 (3)	157
C2—H2⋯O4^ii^	0.95	2.57	3.499 (5)	166
C18—H18⋯O5	1.00	2.57	3.560 (4)	163
C9—H9⋯O4	0.99	2.33	3.309 (4)	164

Symmetry codes: (i) 
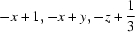
; (ii) 
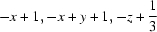
.

**Fig. 1.**
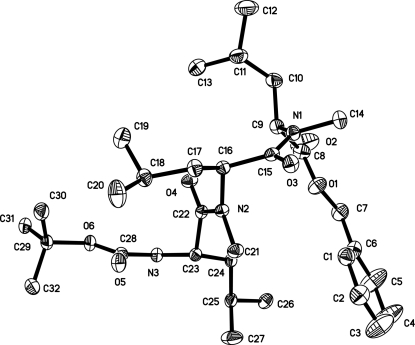
The molecular structure of the title compound with displacement ellipsoids drawn at the 30% probability level.

## supplementary crystallographic information

### Comment

The title compound (I, Fig. 1) was prepared from the dipeptide Benzyl-2-{[2-(*tert*-butoxycarbonylamino)-4-methylpentanoyl]methylamino} -4-methylpentanoate, which is an intermediate product in the synthesis of polypeptides (Xu *et al.*, 2008). The bond lengths and angles of (I) are unexceptional and are in good agreement with the corresponding values in the dipeptide structure (Liao *et al.*, 2007). A classic intermolecular N—H···O hydrogen bond (Table 1) and a weak non-conventional C—H···O contact connect the molecules to form layers parallel to (0 0 1).

### Experimental

The benzyl in Benzyl-2-{[2-(*tert*-butoxycarbonylamino)-4-methylpentanoyl] methylamino}-4-methylpentanoate (2 mmol) was removed, dried and dissolved using THF under an atmosphere of nitrogen. A coupling reagent 3-(diethoxy-phosphoryloxy)-3*H*-benzo[*d*][1,2,3] triazin-4-one (DEPBT 3 mmol) and diisopropylethylamine (DIPEA) were added successively at 273 K. After 10 min, the amine (2.4 mmol) was added in one portion. The mixture was allowed to stand at room temperature for 12 h. The reactions were monitored using TLC. The reaction was concentrated and it was not necessary to carry out the postproccessing, the material was directly subjected to silica gel column chromatography using n-hexane/acetone (20:1) isocratic elution to give the title compound. Colorless crystals suitable for X-ray analysis grew over a period of a week when the solution was exposed to air.

### Refinement

Hydrogen atoms attached to C or N atoms were located at geometrically calculated positions [1.00 Å (CH), 0.99 Å (CH_2_), 0.98 Å (CH_3_), and 0.88 Å (NH)] and refined with isotropic thermal parameters *U*_iso_(H) equal to 1.2 for CH, CH_2_ and NH, and 1.5 for CH_3_*U*_eq_(C atoms).

In the absence of significant anomalous dispersion effects, Friedel pairs were averaged.

### Crystal data

**Table:**

C_32_H_53_N_3_O_6_	*D*_x_ = 1.112 Mg m^−^^3^
*M**_r_* = 575.77	Mo *K*α radiation, λ = 0.71073 Å
Trigonal, *P*3_1_21	Cell parameters from 5764 reflections
Hall symbol: P 31 2"	θ = 2.2–26.7°
*a* = 13.9784 (3) Å	µ = 0.08 mm^−^^1^
*c* = 30.4763 (15) Å	*T* = 173 K
*V* = 5157.1 (3) Å^3^	Block, colorless
*Z* = 6	0.47 × 0.42 × 0.26 mm
*F*(000) = 1884	

### Data collection

**Table:**

Bruker SMART 1000 CCD diffractometer	4232 independent reflections
Radiation source: fine-focus sealed tube	3541 reflections with *I* > 2σ(*I*)
graphite	*R*_int_ = 0.032
ω scans	θ_max_ = 27.1°, θ_min_ = 1.7°
Absorption correction: multi-scan (SADABS; Sheldrick, 1996)	*h* = −17→13
*T*_min_ = 0.965, *T*_max_ = 0.981	*k* = −17→17
27507 measured reflections	*l* = −39→23

### Refinement

**Table:**

Refinement on *F*^2^	Primary atom site location: structure-invariant direct methods
Least-squares matrix: full	Secondary atom site location: difference Fourier map
*R*[*F*^2^ > 2σ(*F*^2^)] = 0.048	Hydrogen site location: inferred from neighbouring sites
*wR*(*F*^2^) = 0.138	H-atom parameters constrained
*S* = 1.05	*w* = 1/[σ^2^(*F*_o_^2^) + (0.076*P*)^2^ + 1.5629*P*] where *P* = (*F*_o_^2^ + 2*F*_c_^2^)/3
4232 reflections	(Δ/σ)_max_ < 0.001
381 parameters	Δρ_max_ = 0.43 e Å^−^^3^
18 restraints	Δρ_min_ = −0.23 e Å^−^^3^

### Special details

**Table:**

Geometry. All e.s.d.'s (except the e.s.d. in the dihedral angle between two l.s. planes) are estimated using the full covariance matrix. The cell e.s.d.'s are taken into account individually in the estimation of e.s.d.'s in distances, angles and torsion angles; correlations between e.s.d.'s in cell parameters are only used when they are defined by crystal symmetry. An approximate (isotropic) treatment of cell e.s.d.'s is used for estimating e.s.d.'s involving l.s. planes.
Refinement. Refinement of *F*^2^ against ALL reflections. The weighted *R*-factor *wR* and goodness of fit *S* are based on *F*^2^, conventional *R*-factors *R* are based on *F*, with *F* set to zero for negative *F*^2^. The threshold expression of *F*^2^ > σ(*F*^2^) is used only for calculating *R*-factors(gt) *etc*. and is not relevant to the choice of reflections for refinement. *R*-factors based on *F*^2^ are statistically about twice as large as those based on *F*, and *R*- factors based on ALL data will be even larger.

### Fractional atomic coordinates and isotropic or equivalent isotropic displacement parameters (Å^2^)

**Table:**

	*x*	*y*	*z*	*U*_iso_*/*U*_eq_	
C1	0.2754 (3)	0.1859 (3)	0.23021 (11)	0.0520 (8)	
H1	0.3538	0.2238	0.2318	0.062*	
C2	0.2238 (4)	0.2413 (3)	0.21488 (13)	0.0598 (10)	
H2	0.2666	0.3165	0.2064	0.072*	
C3	0.1141 (4)	0.1899 (4)	0.2119 (2)	0.0928 (17)	
H3	0.0784	0.2276	0.2010	0.111*	
C4	0.0541 (4)	0.0827 (5)	0.2248 (3)	0.130 (3)	
H4	−0.0242	0.0461	0.2234	0.156*	
C5	0.1050 (4)	0.0259 (4)	0.2399 (2)	0.1001 (19)	
H5	0.0617	−0.0496	0.2480	0.120*	
C6	0.2170 (3)	0.0782 (3)	0.24319 (12)	0.0505 (8)	
C7	0.2703 (3)	0.0129 (3)	0.25971 (12)	0.0519 (8)	
H7A	0.2490	−0.0099	0.2906	0.062*	
H7B	0.2463	−0.0542	0.2416	0.062*	
C8	0.4532 (3)	0.0463 (3)	0.26799 (10)	0.0437 (7)	
C9	0.5736 (3)	0.1334 (2)	0.26120 (9)	0.0360 (6)	
H9	0.5924	0.1264	0.2301	0.043*	
C10	0.6515 (3)	0.1151 (3)	0.29028 (10)	0.0457 (8)	
H10A	0.6264	0.0352	0.2910	0.055*	
H10B	0.6474	0.1384	0.3206	0.055*	
C11	0.7710 (3)	0.1778 (3)	0.27493 (11)	0.0511 (8)	
H11	0.7880	0.2533	0.2656	0.061*	
C12	0.8493 (4)	0.1908 (5)	0.31243 (15)	0.0809 (14)	
H12A	0.8332	0.1177	0.3225	0.121*	
H12B	0.8392	0.2308	0.3367	0.121*	
H12C	0.9259	0.2323	0.3020	0.121*	
C13	0.7895 (4)	0.1226 (5)	0.23608 (13)	0.0694 (12)	
H13A	0.7761	0.0493	0.2447	0.104*	
H13B	0.8658	0.1675	0.2258	0.104*	
H13C	0.7385	0.1149	0.2124	0.104*	
C14	0.5605 (3)	0.2723 (3)	0.30884 (9)	0.0432 (7)	
H14A	0.6278	0.3280	0.3236	0.065*	
H14B	0.5241	0.2056	0.3270	0.065*	
H14C	0.5104	0.3015	0.3045	0.065*	
C15	0.6162 (2)	0.3192 (2)	0.23346 (9)	0.0321 (6)	
C16	0.6491 (2)	0.2951 (2)	0.18839 (8)	0.0298 (5)	
H16	0.6844	0.2488	0.1929	0.036*	
C17	0.7328 (2)	0.4028 (2)	0.16595 (9)	0.0379 (6)	
H17A	0.6977	0.4481	0.1603	0.045*	
H17B	0.7961	0.4449	0.1859	0.045*	
C18	0.7752 (3)	0.3829 (3)	0.12259 (10)	0.0412 (7)	
H18	0.7100	0.3250	0.1061	0.049*	
C19	0.8540 (4)	0.3405 (4)	0.12951 (13)	0.0650 (10)	
H19A	0.8768	0.3262	0.1010	0.097*	
H19B	0.8174	0.2719	0.1465	0.097*	
H19C	0.9191	0.3958	0.1455	0.097*	
C20	0.8274 (4)	0.4870 (4)	0.09480 (13)	0.0723 (13)	
H20A	0.8921	0.5452	0.1100	0.108*	
H20B	0.7736	0.5114	0.0899	0.108*	
H20C	0.8502	0.4716	0.0665	0.108*	
C21	0.4911 (3)	0.2895 (3)	0.14652 (9)	0.0365 (6)	
H21A	0.5140	0.3169	0.1166	0.055*	
H21B	0.5089	0.3517	0.1662	0.055*	
H21C	0.4113	0.2378	0.1471	0.055*	
C22	0.5311 (2)	0.1349 (2)	0.14422 (8)	0.0296 (5)	
C23	0.4365 (2)	0.0770 (2)	0.11090 (8)	0.0294 (5)	
H23	0.4220	0.1331	0.0968	0.035*	
C24	0.3335 (2)	−0.0066 (2)	0.13576 (9)	0.0317 (6)	
H24A	0.3193	0.0325	0.1597	0.038*	
H24B	0.3488	−0.0618	0.1494	0.038*	
C25	0.2289 (2)	−0.0677 (3)	0.10791 (9)	0.0378 (6)	
H25	0.2416	−0.1116	0.0851	0.045*	
C26	0.1327 (3)	−0.1472 (3)	0.13673 (11)	0.0447 (7)	
H26A	0.1177	−0.1054	0.1587	0.067*	
H26B	0.1517	−0.1978	0.1515	0.067*	
H26C	0.0670	−0.1897	0.1185	0.067*	
C27	0.2013 (3)	0.0120 (4)	0.08478 (14)	0.0685 (12)	
H27A	0.1348	−0.0299	0.0669	0.103*	
H27B	0.2632	0.0616	0.0659	0.103*	
H27C	0.1882	0.0555	0.1067	0.103*	
C28	0.5515 (2)	0.0915 (2)	0.05042 (8)	0.0335 (6)	
C29	0.6809 (3)	0.0907 (3)	−0.00321 (9)	0.0383 (6)	
C30	0.7820 (3)	0.1894 (3)	0.01674 (10)	0.0512 (8)	
H30A	0.7653	0.2483	0.0229	0.077*	
H30B	0.8439	0.2164	−0.0039	0.077*	
H30C	0.8020	0.1668	0.0441	0.077*	
C31	0.7080 (3)	0.0009 (3)	−0.01504 (10)	0.0463 (8)	
H31A	0.7347	−0.0194	0.0111	0.069*	
H31B	0.7653	0.0285	−0.0377	0.069*	
H31C	0.6414	−0.0641	−0.0261	0.069*	
C32	0.6356 (3)	0.1225 (3)	−0.04226 (10)	0.0460 (7)	
H32A	0.5678	0.0576	−0.0526	0.069*	
H32B	0.6905	0.1501	−0.0659	0.069*	
H32C	0.6192	0.1803	−0.0336	0.069*	
N1	0.5894 (2)	0.24496 (19)	0.26592 (7)	0.0338 (5)	
N2	0.54989 (18)	0.23237 (18)	0.16121 (7)	0.0288 (5)	
N3	0.46804 (19)	0.02448 (19)	0.07749 (7)	0.0302 (5)	
H3A	0.4344	−0.0479	0.0749	0.036*	
O1	0.38691 (19)	0.08380 (19)	0.25637 (8)	0.0500 (6)	
O2	0.4211 (3)	−0.0461 (2)	0.27986 (13)	0.0891 (11)	
O3	0.61650 (19)	0.40624 (17)	0.23920 (6)	0.0398 (5)	
O4	0.58401 (17)	0.09059 (17)	0.15495 (7)	0.0391 (5)	
O5	0.5840 (2)	0.18927 (17)	0.04605 (7)	0.0438 (5)	
O6	0.59298 (17)	0.03485 (17)	0.02975 (6)	0.0362 (5)	

### Atomic displacement parameters (Å^2^)

**Table:**

	*U*^11^	*U*^22^	*U*^33^	*U*^12^	*U*^13^	*U*^23^
C1	0.0459 (19)	0.056 (2)	0.0467 (17)	0.0203 (18)	0.0047 (15)	0.0046 (15)
C2	0.059 (2)	0.052 (2)	0.067 (2)	0.0271 (19)	−0.0005 (19)	−0.0033 (18)
C3	0.054 (3)	0.055 (3)	0.167 (5)	0.027 (2)	−0.005 (3)	0.003 (3)
C4	0.048 (3)	0.078 (4)	0.262 (8)	0.029 (3)	0.006 (4)	0.030 (4)
C5	0.042 (2)	0.051 (3)	0.198 (6)	0.016 (2)	0.014 (3)	0.022 (3)
C6	0.0374 (17)	0.0439 (19)	0.0607 (19)	0.0131 (15)	0.0018 (15)	−0.0149 (15)
C7	0.0398 (18)	0.0465 (19)	0.0574 (19)	0.0124 (16)	0.0059 (15)	0.0048 (16)
C8	0.0471 (18)	0.0354 (16)	0.0453 (16)	0.0181 (14)	−0.0030 (14)	0.0052 (13)
C9	0.0430 (16)	0.0338 (15)	0.0351 (14)	0.0221 (13)	0.0023 (12)	0.0029 (11)
C10	0.0532 (19)	0.0512 (19)	0.0404 (15)	0.0319 (16)	−0.0035 (14)	0.0060 (14)
C11	0.049 (2)	0.058 (2)	0.0544 (18)	0.0330 (18)	−0.0101 (15)	−0.0005 (16)
C12	0.069 (3)	0.102 (4)	0.082 (3)	0.051 (3)	−0.031 (2)	−0.016 (3)
C13	0.064 (3)	0.108 (4)	0.061 (2)	0.061 (3)	−0.001 (2)	−0.001 (2)
C14	0.0485 (18)	0.0439 (17)	0.0366 (14)	0.0227 (15)	0.0067 (13)	−0.0019 (13)
C15	0.0271 (13)	0.0327 (14)	0.0364 (13)	0.0149 (11)	−0.0039 (11)	−0.0024 (11)
C16	0.0283 (13)	0.0271 (13)	0.0323 (12)	0.0124 (11)	−0.0015 (10)	−0.0013 (10)
C17	0.0357 (15)	0.0317 (15)	0.0385 (14)	0.0110 (13)	0.0037 (12)	−0.0009 (11)
C18	0.0357 (16)	0.0377 (16)	0.0386 (14)	0.0097 (13)	0.0046 (12)	−0.0005 (12)
C19	0.057 (2)	0.085 (3)	0.061 (2)	0.042 (2)	0.0086 (18)	−0.004 (2)
C20	0.082 (3)	0.062 (3)	0.063 (2)	0.028 (2)	0.032 (2)	0.018 (2)
C21	0.0406 (16)	0.0354 (15)	0.0416 (14)	0.0251 (13)	−0.0056 (12)	−0.0046 (12)
C22	0.0279 (13)	0.0303 (13)	0.0304 (12)	0.0144 (11)	0.0036 (10)	0.0015 (10)
C23	0.0293 (13)	0.0294 (13)	0.0310 (12)	0.0157 (11)	0.0008 (10)	−0.0012 (10)
C24	0.0293 (13)	0.0333 (14)	0.0311 (12)	0.0146 (12)	0.0032 (10)	−0.0004 (11)
C25	0.0286 (14)	0.0444 (17)	0.0366 (14)	0.0154 (13)	0.0020 (11)	−0.0058 (12)
C26	0.0306 (15)	0.0423 (17)	0.0557 (17)	0.0141 (14)	0.0047 (13)	0.0026 (14)
C27	0.0367 (18)	0.080 (3)	0.075 (2)	0.0188 (19)	−0.0063 (18)	0.031 (2)
C28	0.0342 (14)	0.0344 (15)	0.0306 (12)	0.0162 (12)	0.0005 (11)	−0.0011 (11)
C29	0.0335 (15)	0.0444 (17)	0.0325 (13)	0.0161 (13)	0.0068 (11)	0.0034 (12)
C30	0.0382 (17)	0.058 (2)	0.0446 (16)	0.0149 (16)	0.0044 (14)	−0.0013 (15)
C31	0.0451 (18)	0.063 (2)	0.0389 (15)	0.0335 (17)	0.0094 (14)	0.0024 (15)
C32	0.0512 (19)	0.053 (2)	0.0362 (15)	0.0282 (17)	0.0038 (14)	0.0045 (14)
N1	0.0375 (13)	0.0324 (12)	0.0321 (11)	0.0179 (11)	0.0019 (10)	0.0012 (9)
N2	0.0291 (11)	0.0259 (11)	0.0325 (10)	0.0146 (9)	−0.0017 (9)	−0.0004 (9)
N3	0.0294 (11)	0.0267 (11)	0.0313 (10)	0.0115 (10)	0.0029 (9)	−0.0022 (9)
O1	0.0342 (12)	0.0387 (13)	0.0714 (15)	0.0139 (10)	0.0029 (11)	0.0066 (11)
O2	0.0613 (19)	0.0446 (16)	0.145 (3)	0.0144 (14)	−0.0121 (19)	0.0392 (18)
O3	0.0495 (13)	0.0329 (10)	0.0424 (10)	0.0247 (10)	0.0024 (9)	−0.0012 (8)
O4	0.0400 (12)	0.0364 (11)	0.0479 (11)	0.0242 (10)	−0.0094 (9)	−0.0083 (9)
O5	0.0502 (13)	0.0315 (11)	0.0461 (11)	0.0176 (10)	0.0135 (10)	0.0055 (9)
O6	0.0374 (11)	0.0361 (11)	0.0362 (9)	0.0191 (9)	0.0109 (8)	0.0039 (8)

### Geometric parameters (Å, °)

**Table:**

C1—C6	1.364 (5)	C18—C20	1.518 (5)
C1—C2	1.377 (6)	C18—H18	1.0000
C1—H1	0.9500	C19—H19A	0.9800
C2—C3	1.331 (6)	C19—H19B	0.9800
C2—H2	0.9500	C19—H19C	0.9800
C3—C4	1.359 (8)	C20—H20A	0.9800
C3—H3	0.9500	C20—H20B	0.9800
C4—C5	1.382 (8)	C20—H20C	0.9800
C4—H4	0.9500	C21—N2	1.473 (3)
C5—C6	1.362 (6)	C21—H21A	0.9800
C5—H5	0.9500	C21—H21B	0.9800
C6—C7	1.524 (5)	C21—H21C	0.9800
C7—O1	1.426 (4)	C22—O4	1.223 (3)
C7—H7A	0.9900	C22—N2	1.355 (3)
C7—H7B	0.9900	C22—C23	1.537 (4)
C8—O2	1.193 (4)	C23—N3	1.447 (3)
C8—O1	1.320 (4)	C23—C24	1.526 (4)
C8—C9	1.520 (5)	C23—H23	1.0000
C9—N1	1.468 (4)	C24—C25	1.530 (4)
C9—C10	1.522 (4)	C24—H24A	0.9900
C9—H9	1.0000	C24—H24B	0.9900
C10—C11	1.522 (5)	C25—C27	1.521 (5)
C10—H10A	0.9900	C25—C26	1.523 (4)
C10—H10B	0.9900	C25—H25	1.0000
C11—C13	1.505 (5)	C26—H26A	0.9800
C11—C12	1.529 (5)	C26—H26B	0.9800
C11—H11	1.0000	C26—H26C	0.9800
C12—H12A	0.9800	C27—H27A	0.9800
C12—H12B	0.9800	C27—H27B	0.9800
C12—H12C	0.9800	C27—H27C	0.9800
C13—H13A	0.9800	C28—O5	1.213 (4)
C13—H13B	0.9800	C28—O6	1.349 (3)
C13—H13C	0.9800	C28—N3	1.351 (4)
C14—N1	1.475 (4)	C29—O6	1.473 (3)
C14—H14A	0.9800	C29—C32	1.516 (4)
C14—H14B	0.9800	C29—C30	1.523 (5)
C14—H14C	0.9800	C29—C31	1.525 (5)
C15—O3	1.227 (3)	C30—H30A	0.9800
C15—N1	1.344 (4)	C30—H30B	0.9800
C15—C16	1.538 (4)	C30—H30C	0.9800
C16—N2	1.470 (3)	C31—H31A	0.9800
C16—C17	1.530 (4)	C31—H31B	0.9800
C16—H16	1.0000	C31—H31C	0.9800
C17—C18	1.529 (4)	C32—H32A	0.9800
C17—H17A	0.9900	C32—H32B	0.9800
C17—H17B	0.9900	C32—H32C	0.9800
C18—C19	1.504 (5)	N3—H3A	0.8800
			
C6—C1—C2	121.7 (4)	C18—C19—H19C	109.5
C6—C1—H1	119.1	H19A—C19—H19C	109.5
C2—C1—H1	119.1	H19B—C19—H19C	109.5
C3—C2—C1	120.4 (4)	C18—C20—H20A	109.5
C3—C2—H2	119.8	C18—C20—H20B	109.5
C1—C2—H2	119.8	H20A—C20—H20B	109.5
C2—C3—C4	119.0 (5)	C18—C20—H20C	109.5
C2—C3—H3	120.5	H20A—C20—H20C	109.5
C4—C3—H3	120.5	H20B—C20—H20C	109.5
C3—C4—C5	121.2 (5)	N2—C21—H21A	109.5
C3—C4—H4	119.4	N2—C21—H21B	109.5
C5—C4—H4	119.4	H21A—C21—H21B	109.5
C6—C5—C4	120.0 (4)	N2—C21—H21C	109.5
C6—C5—H5	120.0	H21A—C21—H21C	109.5
C4—C5—H5	120.0	H21B—C21—H21C	109.5
C5—C6—C1	117.7 (4)	O4—C22—N2	123.0 (2)
C5—C6—C7	118.6 (4)	O4—C22—C23	119.2 (2)
C1—C6—C7	123.7 (3)	N2—C22—C23	117.7 (2)
O1—C7—C6	106.9 (3)	N3—C23—C24	111.8 (2)
O1—C7—H7A	110.3	N3—C23—C22	109.4 (2)
C6—C7—H7A	110.3	C24—C23—C22	108.0 (2)
O1—C7—H7B	110.3	N3—C23—H23	109.2
C6—C7—H7B	110.3	C24—C23—H23	109.2
H7A—C7—H7B	108.6	C22—C23—H23	109.2
O2—C8—O1	123.6 (3)	C23—C24—C25	115.1 (2)
O2—C8—C9	125.1 (3)	C23—C24—H24A	108.5
O1—C8—C9	111.2 (3)	C25—C24—H24A	108.5
N1—C9—C8	110.8 (3)	C23—C24—H24B	108.5
N1—C9—C10	112.4 (3)	C25—C24—H24B	108.5
C8—C9—C10	112.6 (2)	H24A—C24—H24B	107.5
N1—C9—H9	106.9	C27—C25—C26	110.4 (3)
C8—C9—H9	106.9	C27—C25—C24	111.7 (3)
C10—C9—H9	106.9	C26—C25—C24	109.6 (2)
C9—C10—C11	113.3 (3)	C27—C25—H25	108.3
C9—C10—H10A	108.9	C26—C25—H25	108.3
C11—C10—H10A	108.9	C24—C25—H25	108.3
C9—C10—H10B	108.9	C25—C26—H26A	109.5
C11—C10—H10B	108.9	C25—C26—H26B	109.5
H10A—C10—H10B	107.7	H26A—C26—H26B	109.5
C13—C11—C10	111.5 (3)	C25—C26—H26C	109.5
C13—C11—C12	110.7 (3)	H26A—C26—H26C	109.5
C10—C11—C12	110.7 (3)	H26B—C26—H26C	109.5
C13—C11—H11	107.9	C25—C27—H27A	109.5
C10—C11—H11	107.9	C25—C27—H27B	109.5
C12—C11—H11	107.9	H27A—C27—H27B	109.5
C11—C12—H12A	109.5	C25—C27—H27C	109.5
C11—C12—H12B	109.5	H27A—C27—H27C	109.5
H12A—C12—H12B	109.5	H27B—C27—H27C	109.5
C11—C12—H12C	109.5	O5—C28—O6	125.5 (3)
H12A—C12—H12C	109.5	O5—C28—N3	123.8 (3)
H12B—C12—H12C	109.5	O6—C28—N3	110.7 (2)
C11—C13—H13A	109.5	O6—C29—C32	109.3 (2)
C11—C13—H13B	109.5	O6—C29—C30	111.0 (2)
H13A—C13—H13B	109.5	C32—C29—C30	112.5 (3)
C11—C13—H13C	109.5	O6—C29—C31	102.1 (2)
H13A—C13—H13C	109.5	C32—C29—C31	111.6 (3)
H13B—C13—H13C	109.5	C30—C29—C31	109.9 (3)
N1—C14—H14A	109.5	C29—C30—H30A	109.5
N1—C14—H14B	109.5	C29—C30—H30B	109.5
H14A—C14—H14B	109.5	H30A—C30—H30B	109.5
N1—C14—H14C	109.5	C29—C30—H30C	109.5
H14A—C14—H14C	109.5	H30A—C30—H30C	109.5
H14B—C14—H14C	109.5	H30B—C30—H30C	109.5
O3—C15—N1	121.4 (3)	C29—C31—H31A	109.5
O3—C15—C16	119.4 (2)	C29—C31—H31B	109.5
N1—C15—C16	119.1 (2)	H31A—C31—H31B	109.5
N2—C16—C17	111.3 (2)	C29—C31—H31C	109.5
N2—C16—C15	109.7 (2)	H31A—C31—H31C	109.5
C17—C16—C15	110.4 (2)	H31B—C31—H31C	109.5
N2—C16—H16	108.4	C29—C32—H32A	109.5
C17—C16—H16	108.4	C29—C32—H32B	109.5
C15—C16—H16	108.4	H32A—C32—H32B	109.5
C18—C17—C16	112.5 (2)	C29—C32—H32C	109.5
C18—C17—H17A	109.1	H32A—C32—H32C	109.5
C16—C17—H17A	109.1	H32B—C32—H32C	109.5
C18—C17—H17B	109.1	C15—N1—C9	125.7 (2)
C16—C17—H17B	109.1	C15—N1—C14	117.4 (2)
H17A—C17—H17B	107.8	C9—N1—C14	116.5 (2)
C19—C18—C20	111.1 (3)	C22—N2—C16	117.8 (2)
C19—C18—C17	112.1 (3)	C22—N2—C21	123.3 (2)
C20—C18—C17	110.6 (3)	C16—N2—C21	117.6 (2)
C19—C18—H18	107.6	C28—N3—C23	117.0 (2)
C20—C18—H18	107.6	C28—N3—H3A	121.5
C17—C18—H18	107.6	C23—N3—H3A	121.5
C18—C19—H19A	109.5	C8—O1—C7	119.3 (3)
C18—C19—H19B	109.5	C28—O6—C29	119.8 (2)
H19A—C19—H19B	109.5		
			
C6—C1—C2—C3	−0.7 (7)	C22—C23—C24—C25	176.7 (2)
C1—C2—C3—C4	0.8 (9)	C23—C24—C25—C27	−56.8 (4)
C2—C3—C4—C5	−1.4 (12)	C23—C24—C25—C26	−179.5 (2)
C3—C4—C5—C6	1.7 (13)	O3—C15—N1—C9	172.2 (3)
C4—C5—C6—C1	−1.5 (9)	C16—C15—N1—C9	−8.8 (4)
C4—C5—C6—C7	−179.6 (6)	O3—C15—N1—C14	−0.1 (4)
C2—C1—C6—C5	1.0 (6)	C16—C15—N1—C14	178.8 (2)
C2—C1—C6—C7	179.0 (3)	C8—C9—N1—C15	−111.7 (3)
C5—C6—C7—O1	175.1 (4)	C10—C9—N1—C15	121.2 (3)
C1—C6—C7—O1	−2.9 (5)	C8—C9—N1—C14	60.7 (3)
O2—C8—C9—N1	−154.8 (4)	C10—C9—N1—C14	−66.3 (3)
O1—C8—C9—N1	29.2 (4)	O4—C22—N2—C16	9.8 (4)
O2—C8—C9—C10	−27.9 (5)	C23—C22—N2—C16	−172.0 (2)
O1—C8—C9—C10	156.1 (3)	O4—C22—N2—C21	176.2 (3)
N1—C9—C10—C11	−71.4 (3)	C23—C22—N2—C21	−5.5 (4)
C8—C9—C10—C11	162.6 (3)	C17—C16—N2—C22	112.7 (3)
C9—C10—C11—C13	−76.7 (4)	C15—C16—N2—C22	−124.8 (2)
C9—C10—C11—C12	159.6 (3)	C17—C16—N2—C21	−54.5 (3)
O3—C15—C16—N2	−92.8 (3)	C15—C16—N2—C21	68.0 (3)
N1—C15—C16—N2	88.2 (3)	O5—C28—N3—C23	−16.8 (4)
O3—C15—C16—C17	30.3 (4)	O6—C28—N3—C23	162.6 (2)
N1—C15—C16—C17	−148.7 (3)	C24—C23—N3—C28	173.5 (2)
N2—C16—C17—C18	−62.4 (3)	C22—C23—N3—C28	−66.9 (3)
C15—C16—C17—C18	175.5 (2)	O2—C8—O1—C7	2.9 (5)
C16—C17—C18—C19	−73.4 (4)	C9—C8—O1—C7	178.9 (3)
C16—C17—C18—C20	161.9 (3)	C6—C7—O1—C8	−178.8 (3)
O4—C22—C23—N3	−38.3 (3)	O5—C28—O6—C29	−3.9 (4)
N2—C22—C23—N3	143.3 (2)	N3—C28—O6—C29	176.7 (2)
O4—C22—C23—C24	83.5 (3)	C32—C29—O6—C28	−65.8 (3)
N2—C22—C23—C24	−94.8 (3)	C30—C29—O6—C28	58.8 (4)
N3—C23—C24—C25	−62.9 (3)	C31—C29—O6—C28	175.9 (2)

### Hydrogen-bond geometry (Å, °)

**Table:**

*D*—H···*A*	*D*—H	H···*A*	*D*···*A*	*D*—H···*A*
N3—H3A···O3^i^	0.88	2.09	2.923 (3)	157
C2—H2···O4^ii^	0.95	2.57	3.499 (5)	166
C18—H18···O5	1.00	2.57	3.560 (4)	163
C9—H9···O4	0.99	2.33	3.309 (4)	164

Symmetry codes: (i) −*x*+1, −*x*+*y*, −*z*+1/3; (ii) −*x*+1, −*x*+*y*+1, −*z*+1/3.
